# Discovery of thermophilic *Bacillales* using reduced-representation genotyping for identification

**DOI:** 10.1186/s12866-020-01800-z

**Published:** 2020-05-13

**Authors:** Berenice Talamantes-Becerra, Jason Carling, Andrzej Kilian, Arthur Georges

**Affiliations:** 1 Institute of Applied Ecology, University of Canberra, Canberra, ACT 2601 Australia; 2 Diversity Arrays Technology Pty Ltd, Canberra, ACT 2617 Australia

**Keywords:** Bacterial identification, DArTseq, Genotyping-by-sequencing, Great Artesian Basin, Reduced-representation sequencing, Thermophiles

## Abstract

**Background:**

This study demonstrates the use of reduced-representation genotyping to provide preliminary identifications for thermophilic bacterial isolates. The approach combines restriction enzyme digestion and PCR with next-generation sequencing to provide thousands of short-read sequences from across the bacterial genomes. Isolates were obtained from compost, hot water systems, and artesian bores of the Great Artesian Basin. Genomic DNA was double-digested with two combinations of restriction enzymes followed by PCR amplification, using a commercial provider of DArTseq™, Diversity Arrays Technology Pty Ltd. (Canberra, Australia). The resulting fragments which formed a reduced-representation of approximately 2.3% of the genome were sequenced. The sequence tags obtained were aligned against all available RefSeq bacterial genome assemblies by BLASTn to identify the nearest reference genome.

**Results:**

Based on the preliminary identifications, a total of 99 bacterial isolates were identified to species level, from which 8 isolates were selected for whole-genome sequencing to assess the identification results. Novel species and strains were discovered within this set of isolates. The preliminary identifications obtained by reduced-representation genotyping, as well as identifications obtained by BLASTn alignment of the 16S rRNA gene sequence, were compared with those derived from the whole-genome sequence data, using the same RefSeq sequence database for the three methods. Identifications obtained with reduced-representation sequencing agreed with the identifications provided by whole-genome sequencing in 100% of cases. The identifications produced by BLASTn alignment of 16S rRNA gene sequence to the same database differed from those provided by whole-genome sequencing in 37.5% of cases, and produced ambiguous identifications in 50% of cases.

**Conclusions:**

Previously, this method has been successfully demonstrated for use in bacterial identification for medical microbiology. This study demonstrates the first successful use of DArTseq™ for preliminary identification of thermophilic bacterial isolates, providing results in complete agreement with those obtained from whole-genome sequencing of the same isolates. The growing database of bacterial genome sequences provides an excellent resource for alignment of reduced-representation sequence data for identification purposes, and as the available sequenced genomes continue to grow, the technique will become more effective.

## Background

Thermophiles continue to generate interest owing to the thermostability of their enzymes, which have been adapted for use in scientific and industrial processes The proteins of thermophilic bacteria generally exhibit higher thermostability compared to those of mesophiles, in part because they tend to have stronger hydrophobic interactions amongst their amino acids than in other bacteria [1]. The ability to withstand such extreme temperatures has made the enzymes from thermophilic bacteria of particular interest for commercial, industrial and scientific applications [2–5] in areas such as pharmaceutical [6], food [7, 8] and detergent industries [9].

The classic environments in which thermophilic microorganisms occur are primarily geothermal in nature [10]. The Great Artesian Basin in South Australia has the temperature and chemical properties which are suitable for thermophiles [11–13]. Specifically, there are bores in this region with water temperatures of 90 °C or more [14, 15], some of them with open, running bore drains known to contain communities of thermophilic microorganisms [16]. Thermophilic bacteria are also found in other environments such as compost [17–20] and hot water systems [21, 22].

The isolation and discovery of thermophilic bacteria is a continuing area of research interest around the world. The identification of novel thermophilic isolates is now routinely achieved through DNA sequencing methods. Jain et al. (2018), in a high throughput analysis of 90 thousand bacterial genomes, discussed the importance of accurate estimation of genetic relatedness in species delimitation. In this context, ANI (Average Nucleotide Identity) has been considered one of the standard tools for this task. ANI is calculated as the average nucleotide identity from the set of orthologous genes identified between any two genomes. Organisms belonging to the same species are typically considered to show ANI values of ≥95% in pairwise comparisons [23].

Here we aim to assess reduced-representation sequencing as an alternative method for preliminary identification of isolates derived from sampling locations across Australia. A standard approach to the identification of novel bacterial strains or species utilises partial or complete 16S rRNA gene sequence as a preliminary identification method to screen for potentially novel strains or species among a set of isolates. Candidates identified from the 16S rRNA gene sequencing subsequently undergo whole-genome sequencing. The use of 16S rRNA gene sequencing for bacterial identification is well established, although it has two potential limitations: firstly, in some cases it is necessary to attempt more than one set of PCR primers in order to achieve amplification from bacterial genomes of unknown taxonomic affinity, and secondly, the potential for limited resolution of identifications obtained from the 16S rRNA gene sequence. For this study, we have tested a novel approach of reduced-representation sequencing for the first stage identification of bacterial isolates to identify 99 isolates from a variety of thermal sources. Additionally, we have compared the preliminary identification outcomes obtained from 16S rRNA gene sequence and reduced-representation sequencing with identifications derived from whole-genome sequence on a subset of bacterial isolates. Our method used DArTseq™ (Canberra, Australia) [24], one of several available methods for generating representative sequences from the genome. It uses restriction enzyme digestion followed by PCR and Illumina short-read sequencing to amplify and sequence thousands of restriction fragments as genomic representations. DArTseq™ has been successfully used for a broad range of applications, for breeding of plants and animals [25], for assessment of genetic diversity [26–28] and for ecological genetics [29, 30]. This study represents the first usage of DArTseq™ for identification of thermophilic bacterial isolates.

## Results

### *In-silico* analysis of control *E. coli* O157 (EDL 933) IRMM449 certified reference standard

Reduced-representation sequence assays were performed as a control experiment on the reference standard genomic DNA of *E. coli* O157 (EDL 933) IRMM449 [31], with 6 technical replicates for each combination of restriction enzymes. Correct identification results were produced for all assays at the species and strain level using the Currito3.1 DNA Fragment Analysis Software [32] which was developed for this project. The mean genome coverage obtained for each method was 2.64% for *Pst*I with *Hpa*II and 2.34% for *Pst*I with *Mse*I. The mean BLASTn percentage alignment values obtained against the genome sequence of *E. coli* O157 (EDL 933) IRMM449, GenBank accession number CP008957.1 [31] were 99.9915 and 99.9974%, respectively. The average number of restriction fragments obtained in the sequence output for each method was 2433 and 1836 fragments, respectively. Finally, the average nucleotide sequence distance (NSD) value obtained for each method was 0.000103 and 0.000040 respectively. For the *Pst*I with *Hpa*II enzyme combination, the average NSD values showed less than 1 bp of difference per 10,000 bp aligned.

### Isolation of the strains

A total of 99 bacterial isolates were obtained from 27 different sampling sources. Microbial growth results of 31 isolates from hot water systems and commercial composts are shown in Table 1 and microbial growth results of 68 bacterial isolates from artesian bore water and bore drains are shown in Table 2.

**Table 1:** Microbial growth for samples from hot water systems and commercial compost. Incubation temperature was 62.5 °C, culture media LB broth agar

Source	Sample name	Temperature (T°C)	Microbial growth	No. Bacterial isolates
Domestic hot water systems				
	DPS1	60 °C	(+)(A), (+)(B)	2
	DPS2	61.1 °C	(+)(A), (+)(B)	2
	DPS3	62.6 °C	(+)	1
	DPS4	62.6 °C	(+)(A), (+)(B)	2
	DPS5	79.6 °C	(+)(A), (+)(B), (+)(C)	3
	DPS6	57 °C	(+)(A), (+)(B), (+)(C)	3
	DPS7	57.6 °C	(−)	
	HTR	84 °C	(++)	1
	DHW	60 °C	(+)(A), (+)(B)	2
Commercial compost				
	DMW	na	(+)	1
	MPCC	na	(+)	1
	NFOSA	na	(+)(A), (+)(B), (+)(C)	3
	MMBA	na	(+)(A), (+)(B)	2
	MFBB	na	(+)(A), (+)(B), (+)(C), (+)(D)	4
	MPCB	na	(+)	1
	CBSP	na	(+)(A), (+)(B), (+)(C)	3
Total bacterial isolates				31

na = not applicable; (−) = no growth was observed; (+) = growth was observed; (++) = strong growth was observed; (A), (B), (C) name assigned if more than one microorganism was observed

**Table 2:** Microbial growth for water and mud samples from the Great Artesian Basin. Incubation temperature was 62.5 °C. Culture media: LB broth agar (pH 5, pH 6.8, pH 8) and PBT pH 6.0

						LB Broth		PBT	No. Bacterial isolates
Location	Source	Temperature (°C)	pH	Sample name	pH 5.0	pH 6.8	pH 8.0	pH 6.0	
Birdsville	water	98 °C^a^	nd	Birdsville Bore	nd	(+)	(+)	(−)	2
	bore drain	98 °C^a^	nd	Birdsville mud	nd	(+)(A), (+)(B)	(+)	(+)(A), (+)(B), (+)(C)	6
Clifton hills	water	80 °C	8.0	CHfil	(−)	(−)	(+)(A)	(−)	1
	bore drain	80 °C	nd	CHMUD	(+)	(++)	(++)(A), (++)(B)	(++)	4
Mount Gason	water	80 °C	8.0	MtGfil	nd	(+)(A), (+)(B)	(+)(A)	(+)(A)	4
	bore drain	80 °C	nd	Mt.GODS / Mt. GMUD	(+)(A), (+)(B)	(+)(A), (+)(B), (+)(C), (+)(D), (+)(E), (+)(F), (+)(G)	(+)(A), (+)(B), (+)(C),	(+)	13
Mirra Mita	water	79 °C	8.0	MMfil	nd	(+)	(−)	(−)	1
	bore drain	79 °C	nd	MMMUD1	(−)	(+)	(−)	(+)	2
	bore drain	68 °C	nd	MMMUD2	(++)	(++)	(−)	(−)	2
	bore drain	74 °C	nd	MMMUD3	(−)	(++)	(+)(A)	(+)	3
	bore drain	62 °C	nd	MMMUD4	(−)	(+)(A), (+)(B)	(+)(A), (+)(B)	(+)	5
	bore drain	66 °C	nd	MMMUD5	(−)	(+)(A), (+)(B)	(−)	(−)	2
	bore drain	50 °C	nd	MMMUD6	(−)	(+)(A), (+)(B)	(−)	(−)	2
	bore drain	39 °C	nd	MMMUD7	(−)	(++)	(−)	(−)	1
	bore drain	74 °C	nd	MMMUD8	(+)	(−)	(−)	(−)	1
Mungerannie station	water	78 °C	7.2	Mgnhotfil / MgnCCG	(+)	(+)	(+)	(−)	3
	bore drain	60 °C	nd	MCWH	(−)	(+)(A), (+)(B)	(−)	(−)	2
Mulka	soil	38 °C	nd	MR	(−)	(+)(A)	(+)(A)	(−)	2
Kopperamanna	water	60 °C	8.5	Efil	(−)	(+)(A), (+)(B)	(−)	(++)	3
	soil	38 °C	nd	ECO3	(−)	(−)	(−)	(++)	1
Etadunna station	water	77.9 °C	8.5	Kanufil	nd	(+)	(−)	(−)	1
Dulkaninna	water	47.8 °C	8.5	Dulfil	nd	(+)(+)	(−)	(−)	1
Clayton station	water	34 °C	8.5	Clfil	nd	(+)(A), (+)(B)	(−)	(−)	2
	bore drain	34 °C	nd	CLB	(−)	(+)	(−)	(−)	1
Lake Harry	water	46 °C	8.5	LHfil	nd	(−)	(−)	(−)	
	bore drain	46 °C	nd	LHMUD	(−)	(+)(A), (+)(B)	(−)	(+)	3
Total bacterial isolates									68

(^a^) Based on published temperature (Habermehl and Pestov 2002 [14])

nd = not determined; (−) = no growth was observed; (+) = growth was observed; (++) = strong growth was observed; (A), (B), (C) name assigned if more than one microorganism was observed

Sediment and water samples inoculated into culture media showed different growth depending on the source. Sediment samples collected from flowing bore drains from the Great Artesian Basin, showed heavy microbial growth at various pH values ranging from between 5.0 to 8.0. Microbial colonies within the inoculated agar plates sometimes showed differing morphologies, indicating the possible presence of more than one bacterial strain. Colonies with distinct morphologies were isolated individually (Table 2).

### Species identification

**Fig. 1:**
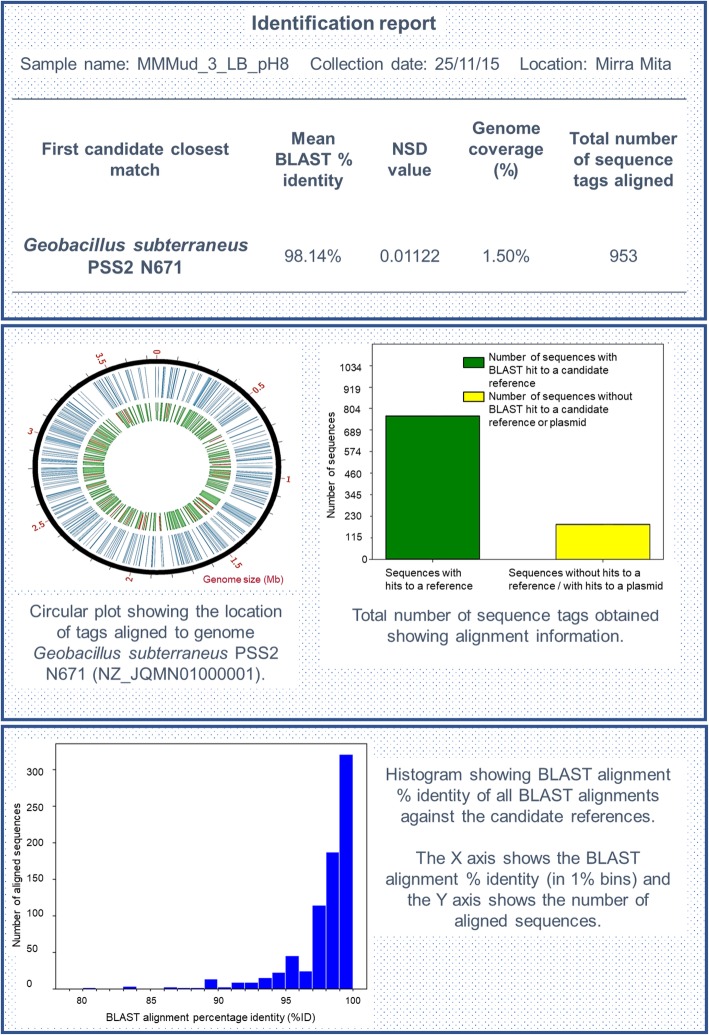
Extract of report generated by bioinformatics pipeline Currito3.1 DNA Fragment Analyser [32] for sample MMMud_3_LB_ph8. This image shows the first candidate closest match to the sample. The circular graph plotted in Circos [33] shows the BLAST alignment position against the reference genome. The outer black circle represents the candidate reference genome with size indicated in megabases (Mb); the middle blue circle shows aligned sequenced fragments obtained by complexity-reduced genotyping and the Inner green / red circle shows the percentage identity of the alignments, in which values below 95% are red and values equal or above 95% are green. The bar plots show the sequences obtained with and without BLAST alignments against the best reference, in which the X axis shows the sequences classification of sequences with and without hits to a reference or plasmid and the Y axis shows the total number of sequences. The histogram shows the percentage identity of BLAST alignments against the candidate reference, where the X axis shows the BLAST alignment percentage identity highest to lowest and the Y axis has the number of aligned sequences

Results obtained from the analysis of the reduced-representation sequences using Currito3.1 DNA Fragment Analysis Software [32] provided preliminary identification and similarity information for all isolates. An example of the report produced by Currito3.1 DNA Fragment Analysis Software [32] for the isolate MMMud_3_LB_pH8 is shown in Fig. 1. The full list of identification results of isolates obtained from hot water systems and commercial composts are shown in Table 3 and the identification results of all isolates from artesian bore water and bore drains are shown in Table 4. Colonies possessing different morphologies within a single plate were isolated and given suffix a, b, c, and d, in some cases these isolates may be duplicates. For each isolate, the nearest sequenced genome from the NCBI RefSeq database is given, along with the average BLASTn percentage identity based on the nearest genome. Percentages obtained ranged from 85.43 to 99.84%. In many instances the BLASTn percentage identity against the nearest genome was > 98.00%, indicating that the isolates belonged to the same species. From this set, 8 isolates were found to have a BLASTn percentage identity against the nearest genome of < 95.00%, indicating potential new species [23].

**Table 3:** Bacterial identification results for isolates obtained from hot water systems and commercial compost, showing nearest matches based on BLAST alignment of complexity-reduced genotyping fragments. Average BLASTn percentage identity, nucleotide sequence distance values to best matches and genome coverage percentage are shown

Source	Sample name	Closest match	Average % identity	Nucleotide sequence distance (NSD)	Genome coverage (%)
Domestic hot water systems					
	DHWa	*Geobacillus sp.* 8	99.27	0.00446	2.28
	DHWb	*Geobacillus sp.* 8	98.84	0.00719	2.12
	DSP1a	*Geobacillus lituanicus* strain N-3	98.18	0.01177	1.80
	DPS1b	*Geobacillus lituanicus* strain N-3	98.11	0.01174	1.80
	DSP2a	*Geobacillus lituanicus* strain N-3	98.58	0.00838	3.25
	DSP2b	*Geobacillus lituanicus* strain N-3	98.58	0.00855	3.23
	DSP3	*Geobacillus lituanicus* strain N-3	98.61	0.00837	3.18
	DSP4a	*Geobacillus lituanicus* strain N-3	98.55	0.00871	3.29
	DSP4b	*Geobacillus lituanicus* strain N-3	98.60	0.00849	3.27
	DSP5a	*Geobacillus lituanicus* strain N-3	98.29	0.01041	4.32
	DSP5b	*Geobacillus lituanicus* strain N-3	98.33	0.01023	4.29
	DPS5c	*Geobacillus lituanicus* strain N-3	98.35	0.01006	4.27
	DSP6a	*Geobacillus lituanicus* strain N-3	98.51	0.00876	3.29
	DSP6b	*Geobacillus sp.* MAS1 T260	98.37	0.00982	2.06
	DSP6c	*Geobacillus sp.* MAS1 T260	98.35	0.00993	2.08
	HTR	*Geobacillus sp.* MAS1 T260	98.29	0.01025	2.02
Commercial compost					
	DMW1	*Geobacillus thermoleovorans* strain ID-1	99.66	0.00189	4.54
	CBSPa	*Geobacillus thermodenitrificans* strain G11MC16	99.39	0.00345	2.03
	CBSPb	*Geobacillus thermodenitrificans* strain G11MC16	99.84	0.00099	2.10
	CBSPc	*Geobacillus thermodenitrificans* strain G11MC16	99.83	0.00105	2.19
	MFBBa	*Geobacillus galactosidasius* strain DSM 18751	98.97	0.00648	1.14
	MFBBb	*Geobacillus thermodenitrificans* strain T12	99.59	0.00248	1.63
	MFBBc	*Geobacillus thermodenitrificans* strain JSC_T9a	99.66	0.00212	1.10
	MFBBd	*Geobacillus galactosidasius* strain DSM 18751	98.65	0.00804	1.14
	MMBAa	*Geobacillus galactosidasius* strain DSM 18751	99.00	0.00671	1.28
	MMBAb	*Geobacillus galactosidasius* strain DSM 18751	98.92	0.00674	1.12
	MPCB	*Geobacillus thermodenitrificans* strain T12	99.58	0.00254	1.59
	MPCC	*Geobacillus thermodenitrificans* strain G11MC16	99.56	0.00267	1.73
	NFOSA1	*Geobacillus thermodenitrificans* strain G11MC16	99.83	0.00106	2.11
	NFOSA2	*Geobacillus sp.* 8	98.69	0.00809	1.85
	NFOSA3	*Geobacillus galactosidasius* strain DSM 18751	98.85	0.00725	1.14

**Table 4:** Bacterial identification results for isolates from the Great Artesian Basin, showing nearest matches based on BLAST alignment of complexity-reduced genotyping fragments. Average BLAST percentage identity, nucleotide sequence distance values to best matches and genome coverage percentage are shown

Location	Sample name	Closest match	Average % identity	Nucleotide sequence distance (NSD)	Genome coverage (%)
Birdsville					
	B_fil_LB_pH6.8_a	*Anoxybacillus ayderensis* strain AB04	98.42	0.00937	2.15
	B_fil_LB_pH6.8_b	*Anoxybacillus ayderensis* strain AB04	98.33	0.00985	2.16
	B_mud_LB_pH6.8_a	*Anoxybacillus suryakundensis* strain DSM 27374	94.92	0.02571	1.43
	B_mud_LB_pH6.8_b	*Anoxybacillus suryakundensis* strain DSM 27374	94.96	0.02496	1.44
	B_mud_LB_pH8	*Geobacillus vulcani* PSS1 N685	99.40	0.00389	2.08
	B_mud_PBT_pH6.0_a	*Anoxybacillus gonensis* strain G2	96.97	0.01787	1.43
	B_mud_PBT_pH6.0_b	*Geobacillus sp.* 8	98.60	0.00858	1.79
	B_mud_PBT_pH6.0_c	*Anoxybacillus kamchatkensis* strain G10	97.78	0.01344	3.01
Clifton hills					
	CHfil_LB_pH8	*Anoxybacillus flavithermus* AK1	99.08	0.00577	3.32
	CHMud_LB_pH5	*Anoxybacillus sp.* 103	98.33	0.00964	1.26
	CHMud_LB_pH6.8	*Geobacillus sp.* 46C-IIa	98.53	0.01127	2.16
	CHMud_LB_pH8	*Anoxybacillus sp.* 103	98.43	0.00903	1.33
	CHMud_PBT_pH6.0	*Anoxybacillus sp.* BCO1 LR68	98.19	0.01060	1.62
Mount Gason					
	MtGfil_LB_pH6.8_a	*Anoxybacillus ayderensis* strain AB04	98.42	0.00951	2.32
	MtGfil_LB_pH6.8_b	*Anoxybacillus sp.* BCO1 LR68	95.49	0.02693	1.88
	MtGfil_LB_pH8	*Anoxybacillus ayderensis* strain AB04	98.21	0.01065	2.36
	MtGfil_PBT_pH6.0	*Anoxybacillus kamchatkensis* strain G10	94.89	0.02937	2.19
	Mt_GMud_LB_pH5	*Anoxybacillus flavithermus* AK1	97.39	0.01570	3.51
	Mt_GMud_LB_pH6.8_a	*Anoxybacillus sp.* BCO1 LR68	97.89	0.01101	2.02
	Mt_GMud_LB_pH6.8_b	*Anoxybacillus kamchatkensis* strain G10	99.34	0.00434	3.45
	Mt_GMud_LB_pH6.8_c	*Anoxybacillus flavithermus* AK1	99.06	0.00561	3.37
	Mt_GMud_LB_pH8	*Anoxybacillus kamchatkensis* strain G10	99.37	0.00424	3.44
	Mt_GMud_PBT_pH6.0	*Anoxybacillus ayderensis* strain AB04	96.60	0.02055	2.48
	Mt_GODS_LB_pH5	*Geobacillus thermoleovorans* strain ID-1	99.01	0.00600	3.60
	Mt_GODS_LB_pH6.8_a	*Anoxybacillus ayderensis* strain AB04	98.41	0.00921	2.18
	Mt_GODSa_LB_pH6.8_b	*Geobacillus thermoleovorans* strain ID-1	97.18	0.01851	2.97
	Mt_GODSb_LB_pH6.8_c	*Anoxybacillus ayderensis* strain AB04	98.36	0.00918	2.31
	Mt_GODSc_LB_pH6.8_d	*Anoxybacillus ayderensis* strain AB04	98.31	0.00937	2.27
	Mt_GODSc_LB_pH8_a	*Anoxybacillus kamchatkensis* strain G10	96.52	0.02288	3.22
	Mt_GODSa_LB_pH8_b	*Anoxybacillus ayderensis* strain AB04	98.25	0.00969	2.33
Mirra Mita					
	MMfil_LB_25/11/15	*Anoxybacillus flavithermus* AK1	98.10	0.01448	3.84
	MMMud_1_LB_pH6.8	*Geobacillus subterraneus* PSS2 N671	98.15	0.01108	1.52
	MMMud_1_PBT_pH6.0	*Geobacillus sp.* MAS1 T260	97.74	0.01651	1.12
	MMMud_2_LB_pH5	*Geobacillus subterraneus* PSS2 N671	96.10	0.02497	1.74
	MMMud_2_LB_pH6.8	*Anoxybacillus kamchatkensis* strain G10	94.41	0.03313	2.31
	MMMud_3_LB_pH6.8	*Geobacillus subterraneus* PSS2 N671	98.06	0.01171	1.55
	MMMud_3_LB_pH8	*Geobacillus subterraneus* PSS2 N671	98.14	0.01122	1.49
	MMMud_3_PBT_pH6.0	*Geobacillus jurassicus* NBRC 107829	99.05	0.00721	1.49
	MMMud_4_LB_pH6.8_a	*Anoxybacillus gonensis* strain G2	99.26	0.00500	2.81
	MMMud_4_LB_pH6.8_b	*Anoxybacillus gonensis* strain G2 AG-1	99.65	0.00358	1.87
	MMMud_4_LB_pH8_a	*Anoxybacillus gonensis* strain G2 AG-1	99.49	0.00417	1.87
	MMMud_4_LB_pH8_b	*Anoxybacillus ayderensis* strain AB04	98.41	0.00908	2.25
	MMMud_4_PBT_pH6.0	*Geobacillus subterraneus* PSS2 N671	98.10	0.01148	1.38
	MMMud_5_LB_pH6.8_a	*Geobacillus thermoleovorans* strain ID-1	99.60	0.00221	3.60
	MMMud_5_LB_pH6.8_b	*Geobacillus sp.* 8	99.29	0.00443	2.39
	MMMud_6_LB_pH6.8_a	*Geobacillus thermoleovorans* strain ID-1	99.25	0.00438	3.58
	MMMud_6_LB_pH6.8_b	*Geobacillus kaustophilus* strain Et7/4 LG52	85.43	0.09046	8.38
	MMMud_7_LB_pH5	*Geobacillus thermoleovorans* strain ID-1	95.90	0.02818	2.53
	MMMud_8_LB_pH5	*Geobacillus vulcani* PSS1 N685	97.59	0.01704	2.12
Mungerannie station					
	MgnHotfil_LB_pH6.8	*Geobacillus vulcani* PSS1 N685	99.40	0.00403	1.88
	Mgn_CCG_LB_pH5	*Anoxybacillus gonensis* strain G2 AG-1	95.60	0.02500	1.68
	Mgn_CCG_LB_pH8	*Anoxybacillus flavithermus* AK1	98.93	0.00522	3.54
	MCWH_LB_pH6.8	*Anoxybacillus flavithermus* AK1	99.04	0.00473	3.52
	MCWH_LB_pH8	*Brevibacillus thermoruber* PM1 N690	94.13	0.06061	2.92
Mulka					
	MR_LB_pH6.8	*Geobacillus kaustophilus* GBlys	99.86	0.00100	1.73
	MR_LB_pH8	*Geobacillus thermodenitrificans* strain OS27	99.64	0.00212	1.41
Kopperamanna					
	Efil_LB_pH6.8_a	*Anoxybacillus ayderensis* strain AB04	98.40	0.00959	2.30
	Efil_LB_pH6.8_b	*Geobacillus thermoleovorans* strain ID-1	95.82	0.02610	2.71
	Efil_PBT_pH6.0	*Anoxybacillus kamchatkensis* strain G10	94.90	0.02852	2.23
	ECO3_PBT_pH6.0	*Geobacillus thermoleovorans* strain KCTC 3570	98.08	0.01121	2.05
Etadunna station					
	Kanufil_LB_pH6.8	*Geobacillus vulcani* PSS1 N685	99.39	0.00394	1.94
Dulkaninna					
	Dufil_LB_pH6.8	*Anoxybacillus kamchatkensis* strain G10	94.92	0.02867	2.23
Clayton station					
	Clfil_LB_pH6.8	*Anoxybacillus ayderensis* strain AB04	98.43	0.00933	2.30
	Clfil_LB_pH6.8	*Anoxybacillus ayderensis* strain AB04	94.36	0.03240	2.29
	CLB_LB_pH6.8	*Anoxybacillus sp.* BCO1 LR68	97.34	0.01678	1.30
Lake Harry					
	LH_Mud_LB_pH6.8_a	*Anoxybacillus gonensis* strain G2 AG-1	99.57	0.00379	1.88
	LH_Mud_LB_pH6.8_b	*Geobacillus jurassicus* NBRC 107829	98.34	0.01092	1.73
	LH_Mud_PBT_pH6.0	*Geobacillus thermoleovorans* strain ID-1	99.04	0.00608	3.57

A total of 16 bacterial isolates were collected from nine domestic hot water systems. Temperatures at which these were collected ranged between 60 °C to 84 °C. A total of 15 bacterial isolates were obtained from seven commercial garden compost sources. The composting temperatures were not recorded, although the range of temperatures occurring during the high-temperature phase of the composting process has been reported as between 40 °C to 78 °C [34]. All isolates derived from omestic hot water systems and compost were identified as belonging to the genus *Geobacillus*.

A total of 18 bacterial isolates were obtained from filtered water samples of artesian bores in The Great Artesian Basin. The range of temperatures at which the water samples were collected was between 34 °C to 98 °C. The pH values ranged from 7.2 to 8.5. From the total, 15 bacterial isolates belonged to the genus *Anoxybacillus* and three isolates belonged to the genus *Geobacillus*.

A total of 50 bacterial isolates were obtained from artesian bore drains in the Great Artesian Basin. Artesian bore drain sediments produced the greatest diversity of strains in this study. The temperatures at which sediment was collected ranged between 34 °C to 98 °C. A total of 26 bore drain bacterial isolates belonged the genus *Anoxybacillus*, 23 isolates belonged to the genus *Geobacillus* and one isolate belonged to the genus *Brevibacillus*.

### Whole-genome sequencing

To test the accuracy of preliminary identifications produced by reduced-representation sequencing, eight samples were selected for whole-genome sequencing. The eight genome assemblies were chosen to form three groups, A, B and C, based on the similarity to the nearest sequenced reference, as determined by the reduced-representation sequence analysis. Group A included isolates for which the identifications showed high similarity (99.4–99.64%) to existing sequenced genomes. Group B included isolates with moderate relatedness (98.14–98.85%) to their nearest identified genome assemblies, and group C contained isolates more distantly related (< 94.13%) to any of the sequenced genome assemblies, representing potential new species. The assembly statistics for the draft genomes of the eight bacterial isolates are shown in Table 5. The identification results obtained from progressiveMauve [35, 36] alignment of the eight draft genomes were compared with those derived from the reduced-representation sequence tags, and with the identifications based on 16S rRNA gene sequence alignments. These results are shown in Table 6. The results presented in this table show that identifications obtained with reduced-representation sequencing agreed with the identifications provided by whole-genome sequencing in 100% of cases. The identifications produced by BLASTn alignment of 16S rRNA gene sequence to the same database differed from those provided by whole-genome sequencing in 37.5% of cases, and produced ambiguous identifications in 50% of cases.

**Table 5:** Whole genome sequencing assembly statistics and nearest relative based on progressiveMAUVE [35, 36]

Group	Sample name	GenBank accession	Reads	contigs	Largest contig	Total length	N50	GC (%)	Nearest relative based on MAUVE
**A**	MR_LB_pH8	SDLB00000000	3,295,260	153	166,175	3,592,399	61,995	48.89	*Geobacillus thermodenitrificans* strain KCTC3902
	B_mud_LB_pH8	SDLA00000000	275,247	60	765,875	3,434,851	188,577	52.02	*Geobacillus vulcani* PSS1
**B**	NFOSA3	SDLE00000000	2,067,951	111	273,724	3,334,687	72,143	42.13	*Geobacillus galactosidasius* strain DSM 18751
	DSP4a	SDLD00000000	885,071	188	271,123	3,273,238	58,160	52.32	*Geobacillus lituanicus* strain N-3
	CHMud_LB_pH8	SDLG00000000	636,456	67	226,012	2,712,590	137,053	41.81	*Anoxybacillus sp.* 103
	Efil_LB_pH6.8	SDLH00000000	777,211	53	596,376	2,794,302	321,229	41.90	*Anoxybacillus ayderensis* strain AB04
	MMMud_3_LB_pH8	SDLC00000000	1,174,246	1196	233,455	4,372,943	60,623	55.27	*Geobacillus subterraneus* PSS2
**C**	MCWH_LB_pH8	SDLF00000000	1,349,927	124	345,578	3,934,072	177,957	56.20	*Anoxybacillus flavithermus* strain B4168

**Table 6:** Comparison of bacterial identification methods showing percentage identity for complexity-reduced genotyping based on BLASTn alignment; whole-genome sequencing best matches using progressiveMauve [35, 36] alignment tool; and the best matches obtained with 16S rRNA gene alignment, including multiple results per sample with equal highest bitscore and percentage identity

Group	Sample name	Complexity-reduced genotyping best match	% ID	Whole genome sequencing best match result	Mauve mean similarity profile value	16 s rRNA in silico best match result	% ID
**A**	MR_LB_pH8	*Geobacillus thermodenitrificans* strain OS27	99.64	*Geobacillus thermodenitrificans* strain KCTC3902	13,618.31	*Geobacillus thermodenitrificans* strain KCTC3902	100.00
						*Geobacillus sp.* PA-3 GEPA3	100.00
						*Geobacillus thermodenitrificans* NG80–2	100.00
	B_mud_LB_pH8	*Geobacillus vulcani* PSS1	99.40	*Geobacillus vulcani* PSS1	12,550.22	*Geobacillus vulcani* PSS1	100.00
						*Geobacillus sp.* FW23	100.00
**B**	NFOSA3	*Geobacillus galactosidasius* strain DSM 18751	98.85	*Geobacillus galactosidasius* strain DSM 18751	8003.04	*Geobacillus galactosidasius* strain DSM 18751	100.00
	DSP4a	*Geobacillus lituanicus* strain N-3	98.55	*Geobacillus lituanicus* strain N-3	4299.05	*Geobacillus stearothermophilus strain* FHS-PHGT51	100.00
						*Geobacillus stearothermophilus strain* DSM 458	100.00
						*Geobacillus stearothermophilus strain* GS27	100.00
						*Geobacillus sp.* Sah69	100.00
						*Geobacillus stearothermophilus* ATCC 12980	100.00
						*Geobacillus stearothermophilus* ATCC 7953	100.00
	CHMud_LB_pH8	*Anoxybacillus sp.* 103	98.43	*Anoxybacillus sp.* 103	7800.76	*Anoxybacillus kamchatkensis* strain G10	100.00
	Efil_LB_pH6.8_a	*Anoxybacillus ayderensis* strain AB04	98.40	*Anoxybacillus ayderensis* strain AB04	10,221.31	*Anoxybacillus kamchatkensis* strain G10	99.66
	MMMud_3_LB_pH8	*Geobacillus subterraneus* PSS2	98.14	*Geobacillus subterraneus* PSS2	3684.00	*Geobacillus icigianus* strain G1w1	99.76
						*Geobacillus subterraneus* PSS2 N671	99.76
**C**	MCWH_LB_pH8	*Brevibacillus thermoruber* PM1 N690	94.13	*Brevibacillus thermoruber* 423	3758.04	*Brevibacillus thermoruber* PM1 N690	99.35

## Discussion

The results showed a complete agreement of the progressiveMauve [35, 36] whole-genome identifications and those obtained from the reduced-representation sequence alignments for all eight bacterial isolates. ProgressiveMauve [35, 36] can perform comparative genome alignment of two or more genomes, identifying and aligning conserved genomic DNA regions. ProgressiveMauve [35, 36] identifies locally colinear blocks (LCBs), which are blocks of unbroken sequence homology between genomes. The progressiveMauve [35, 36] algorithm uses an iterative process to identify and refine the boundaries of LCBs identified between genomes. ProgressiveMauve [35, 36] alignment is able to recognize homologous regions in the presence of multiple complex rearrangements and provides a valuable tool for analysis of sequence homology between species and strains [35, 36]. Similarity profile values, corresponding to the average level of nucleotide sequence conservation within regions of local alignment, are calculated by progressiveMauve [35, 36] to be inversely proportional to the average alignment column entropy within the region [35]. To determine a pairwise similarity value between genomes, each of the eight genome assemblies was aligned against the complete set of available RefSeq *Anoxybacillus Geobacillus* and *Brevibacillus* assemblies in a pairwise manner. The mean similarity profile value from each progressiveMauve [35, 36] pairwise alignment was calculated. These values provide a comparative measure of the similarity of each pair of genomes, averaged over all aligned sequence regions between the genome pair.

In each case the same species was identified as the closest match, and in seven of eight cases, the same assembly was identified. For the isolate MCWH_LB_pH8 the two methods identified different assemblies of *Brevibacillus thermoruber*. In all eight cases the progressiveMauve [35, 36] whole-genome identifications and the reduced-representation sequence results identified a single closest matching candidate assembly. The identification results from the 16S rRNA gene sequences sometimes identified multiple closest matching assemblies of equal bitscore and percentage identity. Out of the eight isolates, three did not produce the same species identification results between progressiveMauve [35, 36] and 16S rRNA gene alignment. Additionally, four out of the eight isolates did not identify a single best candidate assembly, based on bitscore and percentage identity. The genome sequences showed that the best 16S rRNA gene alignment did not always match the results from whole-genome sequencing; however, misalignments may indicate an intensive horizontal gene transfer or genome rearrangements rather than phylogenetic diversity.

The sequence fragments produced by reduced representation sequencing represented coverage of approximately 2.3% of the genome, derived from up to 2500 individual fragments, depending on the combination of restriction enzymes used. This number of fragments can be sequenced to a read depth of 40x using 100,000 reads per assay, and the sequence barcoding system allows for multiplexing of up to 2300 assays. The volume of sequencing required to achieve full coverage of the reduce-representation fragments is very low in comparison to the sequencing output of current next-generation sequencers. A single lane of a HiSeq 2500 v4 flow-cell could be used to sequence assays for 2300 isolates with a read depth of >40x across the available fragments. Alternatively, for lower throughput processing, 250 assays could be processed in a MiSeq v3 flow-cell at a 40x read depth. The assay cost per sample, including library construction and sequencing would be expected to be 7 dollars (USD) [37].

### Comparison of identification methods

DNA sequence-based bacterial identification has relied almost exclusively on partial or complete 16S ribosomal RNA gene sequencing [38–42]. In spite of the ubiquitous use of 16S sequence data, the limitations of this approach are well established [43]. One of the first problems identified with this technique was the difficulty of primer design, necessitating attempts at creation of ‘universal’ primers, ideally capable of amplifying a portion of the 16S rRNA gene from any bacterial isolate [44]. In practice, multiple primer pairs may sometimes need to be trialled to obtain successful amplification from any given isolate. Another issue relates to the limited resolution of the identification information provided by 16S rRNA gene sequencing [43]. The original rationale for the choice of the 16S rRNA gene for use in bacterial identification is based on the need for a balance between sequence conservation versus sequence diversity. Sequence similarity between taxa must be sufficient for priming and PCR amplification, but sequence variability must be sufficient to provide resolution between taxa for identification purposes. In practice the resolution provided by 16S rRNA sequence data can be insufficient for species delimitation as they may be identical between species [45]. Effectively, the sequence similarity of the 16S locus may not be a surrogate for the similarity of the genome as a whole [46]. Obtaining a whole-genome sequence is clearly the best option to identify bacterial isolates and determine their nearest relatives; however, the costs involved mean that it is generally not practical to do this for all of bacterial isolates in a study.

An alternative identification method of sequencing complexity-reduced genomic representations could provide a potential replacement, avoiding some of the limitations. This study has shown that reduced-representation sequencing can provide fine scale identification information, most importantly, with complete agreement to whole-genome sequence information in terms of identification for the bacterial isolates tested in this study. Reduced-representation sequences can be produced for any organism, without need for prior sequence information, and with no prior knowledge of taxonomic affinities required.

## Conclusions

This study clearly demonstrates the accuracy of the identifications based on reduced-representation sequencing. The eight isolates were selected for whole-genome sequencing to test the accuracy of the preliminary identifications. In each case the closest matching genomes identified by reduced-representation sequencing agreed completely with the identifications provided by whole-genome sequencing. The identifications provided by 16S rRNA gene sequence alignment, were in agreement for some of the isolates but differed from the whole-genome-based results for others. The 16S rRNA gene results also identified multiple accessions or even multiple species with equal distance in 50% of cases, failing to identify a single best candidate from the sequence database. The method of reduced-representation sequencing has been successfully applied in identification of bacterial isolates in a medical microbiology context [37] and this is the first successful use for identification of thermophilic bacterial isolates.

The genome coverage obtained in this study ranged between 1.10 to 4.54%. This coverage is derived from short fragments obtained from across the entire genome, rather than longer consecutive regions, as shown in the circular graph plotted by Circos [33] from Fig. 1. This potentially allows detection of horizontal transfer between taxa; however, in practice many horizontal gene transfer events may be undetectable in the results produced from this method. Further work will need to be done to clarify the limits of horizontal gene transfer detection and the implications for bacterial identifications.

Reduced-representation sequencing is equally well suited for use with small numbers of isolates or with large batch processing of thousands of assays. The growing database of available bacterial genome sequences provides an excellent resource for alignment of reduced-representation sequences for identification purposes, and as the available genomes continue to grow, the technique will become more effective with time.

## Materials and methods

### Sampling

#### Hot water systems

Water samples were collected from nine domestic hot water systems with at least five years of operation in the region of the ACT, Australia. A volume of 1.5 L was collected into sterile containers, temperature was recorded, and bottles were transported without refrigeration. The water samples were filtered with sterile membranes of 0.20 μm pore size (Nalgene™ Rapid-Flow™, PES Membrane Cat. No. 6.302336, type 565, ThermoFisher Scientific, Australia), connected to a vacuum pump, to capture any bacteria present.

#### Commercial composts

Samples were collected from seven commercial composts. The composts were made from various combinations of animal manure and plant matter. Samples taken from bagged compost were placed in 50 ml sterile falcon tubes and transferred to the laboratory for inoculation.

#### Artesian bores in the great Artesian Basin, South Australia

**Fig. 2:**
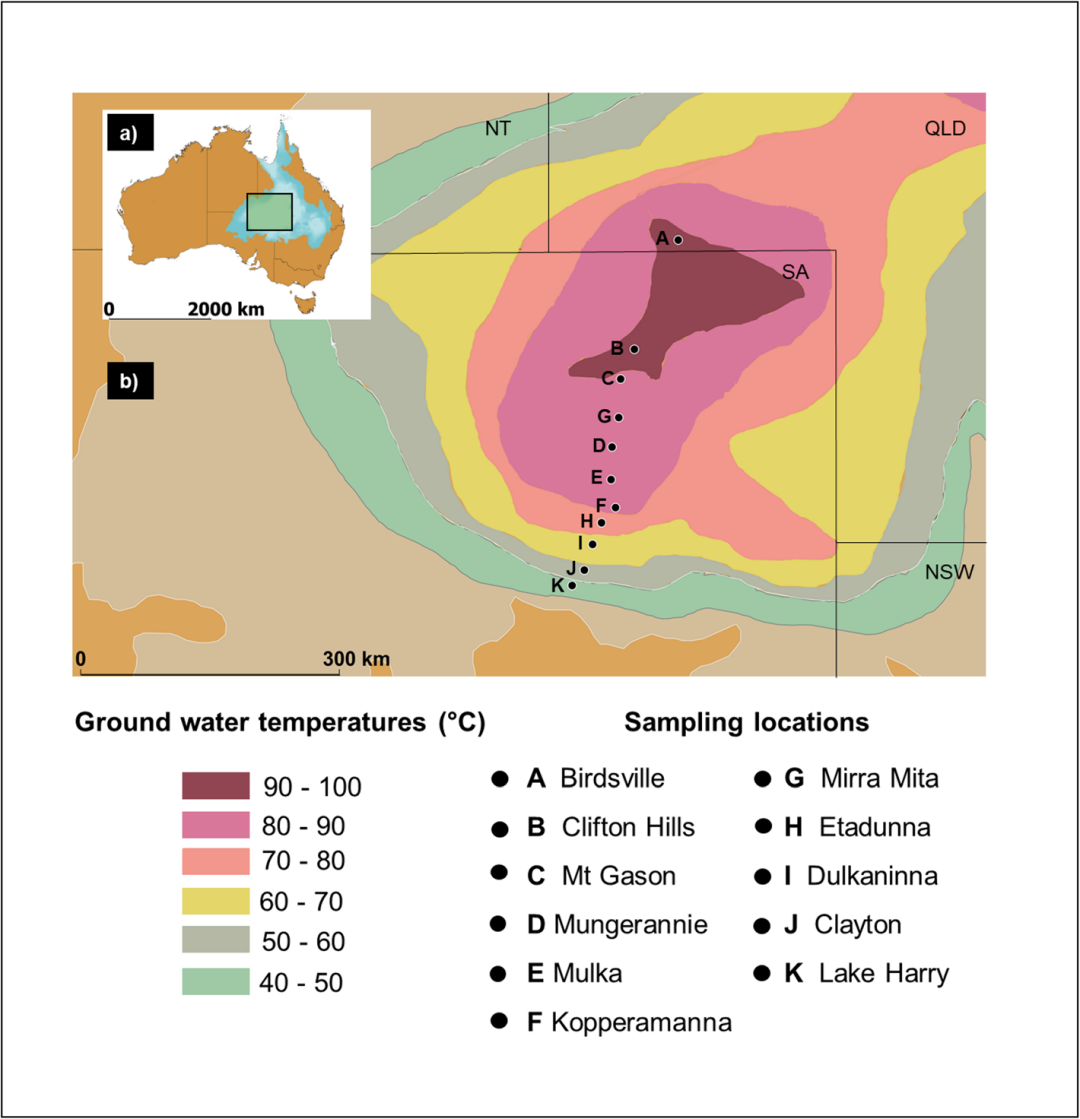
Sampling locations of 11 water-bores of The Great Artesian Basin showing groundwater temperatures derived from Habermehl and Pestov (2002) [14]. Inset map shows larger position of larger map within Australia

A total of 10 water samples were collected from the tap at the bore head into sterile bottles from selected bore locations on the Birdsville Track, South Australia (Fig. 2 and Supplementary Material 1). A volume of 1.5 L was taken, temperature was recorded, and water samples were filtered to collect any bacteria present, using the same method described above for hot water systems. In addition, sediment samples were collected from each of the bore drains at various distances from the bore head along the temperature gradient, especially where colonies of microorganisms were visible. These were collected into 50 ml sterile falcon tubes.

### Medium composition and cultivation

Four types of solid culture media were prepared using two different nutrient recipes and a series of pH values. The first nutrient recipe contained LB medium, and was prepared by dissolving 20 g Lb Broth (Sigma-Aldrich L3022) and 5 g Gelzan™ CM Gelrite® solidifying agent (G1910 Sigma-Aldrich) in 900 ml of miliQ water, then filling up with miliQ water to 1000 ml. Culture media were adjusted for final pH values of 5.0, 6.8 and 8.0. The second nutrient recipe combined 1.0 g L^− 1^ yeast extract, 1.0 g L^− 1^ tryptone, with a basal medium containing 1.3 g (NH_4_)_2_SO_4_, 0.47 g K_2_HPO_4_^.^3H_2_O, 0.25 g MgSO_4_^.^7H_2_O, 0.07 g CaCl_2_ and 1 ml of trace element solution [47]. This was prepared by dissolving all components with 5 g Gelzan™ CM Gelrite® solidifying agent in 900 ml of miliQ water, then filling up with miliQ water to 1000 ml and adjusting to pH 6.0. All media were autoclaved at 121 °C for 20 min.

The four variations of culture media and pH were inoculated with each of the compost, filter strips, and sediment samples, and all cultures were incubated at 62.5 °C. Filter membranes were removed from the filter, cut into strips with a sterile scalpel blade, and placed inverted on the surface of the solid media. Similarly, approximately 1 g of compost/sediment was scattered on the surface of the media. Humidity conditions for the culture plates were controlled by adding sterile wet gauzes inside of a sealed plastic box that contained all Petri dishes. Bacterial growth was observed within a range of time from 48 to 72 h. Individual colonies identified were isolated by at least three passages of subculturing from single cell derived colonies.

### Library preparation and sequencing

DNA extractions were performed for all bacterial isolates using the chloroform-isoamyl alcohol method [37]. The library preparation was done following the DArTseq™ methods, in which the DNA was digested with pairs of restriction enzymes, in this case, *Pst*I with *Hpa*II and *Pst*I with *Mse*I respectively, and PCR adapters were ligated to the fragments. Two adapters were used, one corresponding to each restriction enzyme. The adapter design included Illumina flow-cell specific sequences required for bridge PCR in cluster generation, as well as a barcode region to enable sample multiplexing. The adapters were designed such that only fragments with differing restriction sites at each end were capable of cluster generation. Digestion/ligation was followed by PCR amplification according to Georges et al. (2018). Final PCR products were stored at 4 °C. A post PCR quality control was performed by agarose gel electrophoresis. An equal volume of PCR product from each sample was pooled and then purified with a QIAGEN QIAquick PCR Purification Kit Cat. ID: 28106 (QUIAGEN, Chatstone, Victoria, Australia). For each sample, libraries made with both complexity-reduction methods were pooled together for sequencing. Clustering was done according to Illumina HiSeq SR Cluster Kit V4 recipe v9.0 and HiSeq SR Flow Cell v4 (Illumina Inc., San Diego CA, US). For sequencing, the Flow Cell was loaded according to the Illumina protocols on a HiSeq 2500 sequencer, using HiSeq SBS kit v4 for a total of 77 cycles [30].

A control was also sequenced, using the genomic DNA of *Eschericha coli* O157 (EDL 933) IRMM449 Sigma-Aldrich (Castle Hill, NSW, Australia) certified reference standard, GenBank [47] accession number AE005174.2, genome size of 5,639,399 bp [31]. This control was processed for library construction, sequencing and analysis using methods identical to those used for all other bacterial isolates.

### Data analysis

Data from the sequencer in the form of FastQ files was processed according to the methods described in Talamantes-Becerra et al. (2019). Briefly, sequences were filtered by PHRED quality score, barcode sequences were removed and identical sequences were recognised and collapsed into ‘fastQcol’ files, which contain each unique sequence present in the original FastQ file, along with the respective read counts and the mean quality score at each base [48]. The reverse adapters which were present on sequences derived from fragments shorter than 69 bp were identified and trimmed, resulting in sequences from 30 bp to 69 bp.

Data for each of the complexity reduction methods was processed with the analytical pipeline Currito3.1 DNA Fragment Analysis Software [32], which we developed specifically for analysing reduced-representation sequences from bacteria. The details of this software pipeline are described in Talamantes-Becerra et al. (2019). Briefly, a BLASTn alignment [49] of the trimmed sequence tags from each sample against all complete bacterial genome assemblies from the NCBI RefSeq database is performed, to identify the best candidate bacterial genomes for each sample. The BLASTn parameters were used were: word size 12, bitscore 50, evalue 0.000001, percentage identity 80, percentage query cover 80%. Candidate matching genome assemblies are selected according to the number of sequence tags obtaining a best or equal best BLASTn hit to each reference, as measured by bit score. After identifying candidate genomes, the trimmed sequences from each sample are aligned by BLASTn against the top three closest identified genomes individually. The Currito3.1 [32] pipeline uses the NSD calculation, shown in the following equation to determine the best matching candidate genome for each sample, based on the BLASTn alignments described above.

$$ NSD=-\frac{3}{4}\ \ln \left[1-\frac{4}{3}\left(\frac{S}{I+S}\right)\right]\left[1-\frac{G}{T}\right]+\frac{G}{T} $$

NSD is a DNA sequence distance measurement considering identities (I), substitutions (S) and gap openings (G) across all aligned sequences to produce a global distance value [50]. Closer relatedness to a reference genome is associated with lower NSD values.

### Confirmation of strain identification results by whole-genome sequencing

The genomic DNA of selected isolates was purified using the Agencourt AMPure XP (Beckman Coulter Inc., Brea, CA, US) genomic DNA purification beads protocol. The volume of Agencourt AMPure XP beads used for purification was 0.4 x genomic DNA volume. Briefly, genomic DNA clean-up was done as follows: selected volumes of AMPure XP beads and genomic DNA was mixed by pipette, then incubated for 5 min at room temperature. Sample tubes were placed onto the magnetic plate for 2 min, and supernatant was discarded carefully leaving 5 μL. Then 200 μL of freshly prepared 70% ethanol was added to the beads, incubating for 30 s before removal of the supernatant over the magnetic plate. This wash step was repeated. After the second wash, ethanol was completely removed and beads were allowed to dry. Samples were removed from the magnetic plate, eluted in 35 μL of EB buffer (10 mM Tris-Cl, pH 8.5), and mixed by pipette 10 times. Sample tubes were located again onto the magnetic plate for 1 min and elution buffer was transferred into a new tube.

The whole-genome sequencing service was provided by MicrobesNG, IMI – School of Biosciences, University of Birmingham, United Kingdom. Libraries for whole-genome sequencing were prepared with the Nextera XT Library Prep Kit (Illumina, San Diego, USA), then quantified with the Microlab STAR automated liquid handling system. All libraries were quantified and pooled for sequencing with the Kapa Biosystems Library Quantification Kit for Illumina on a Roche light cycler 96 qPCR machine and were sequenced on the Illumina HiSeq2500 using a 250 bp paired end protocol. Sequencing depth for all bacterial isolates was 30X. To process the sequence data, Trimmomatic 0.30 [51] was used for trimming reads, SPAdes version 3.7 [52] was used for de novo assembly, and Prokka 1.11 [53] was used for annotation of contigs.

The resulting draft genome assemblies for the 8 isolates were assessed using the software tool Kraken [54], which utilises DNA sequence K-mer alignments to determine taxonomic affinities. The Kraken results placed all isolates within the same genera reported by the Currito3.1 [32] analytical pipeline.

The draft genome assemblies were then aligned against all available *Anoxybacillus*, *Geobacillus* and *Brevibacillus* assemblies totalling 31, 72 and 63 genome assemblies respectively, from GenBank [55] database up to December 2018, to identify the closest matching genome assembly for each isolate. For alignment of the whole-genome assemblies, the software progressiveMauve [35, 36] was used.

For each of the eight draft genomes, the pairwise alignment which resulted in the highest mean similarity profile value was considered the closest matching genome. The identifications obtained in this way were used to test the accuracy of the identifications obtained using the method under current investigation, based on reduced-representation sequencing. In addition to the genome alignments, the complete 16S rRNA gene CDS sequence was also used to identify the closest genome assembly from amongst the same set of available RefSeq [55] assemblies. For each of the eight isolates, the 16S rRNA gene sequence was obtained from the draft genome assembly using the annotation produced by Prokka 1.11 [53]. The complete 16S rRNA gene sequences were aligned by BLASTn against each of the RefSeq *Anoxybacillus*, *Geobacillus* and *Brevibacillus* genome assemblies in order to find the best or equal best matches for the 16S rRNA gene, along with percentage identity values.

## Supplementary information

**Additional file 1.**

## Data Availability

Scripts for Currito3.1 DNA Fragment Analysis Software are available on GitHub. https://github.com/BTalamantesBecerra/Currito3.110.5281/zenodo.3748447 The datasets generated and/or analysed during the current study are available on Figshare Repository. DOI: 10.6084/m9.figshare.11930892 This Whole-Genome Shotgun project has been deposited at DDBJ/ENA/GenBank under the accessions SDLA00000000, SDLB00000000, SDLC00000000, SDLD00000000, SDLE00000000, SDLF00000000, SDLG00000000, SDLH00000000. The raw FASTQ reads have been deposited in the NCBI SRA database under the accession numbers SRR8490233, SRR8490232, SRR8490231, SRR8490230, SRR8490229, SRR8490228, SRR8490227, SRR8490226.

## Abbreviations

16S rRNA: 16S ribosomal RNA
ACT: Australian Capital Territory
ANI: Average Nucleotide Identity
BLASTn: Basic Local Alignment Search Tool (Nucleotides)
*E. coli*: *Escherichia coli*
LCBs: Locally Colinear Blocks
NCBI: National Center for Biotechnology Information
NSD: Nucleotide Sequence Distance
PCR: Polymerase Chain Reaction
