# Supplementary material for: Discovery of thermophilic Bacillales using reduced-representation genotyping for identification
Source: BMC Microbiol. 2020 May 13;20:114. doi: 10.1186/s12866-020-01800-z (PMC7222431; doi:10.1186/s12866-020-01800-z)
Supplement: Supplementary file 1 — Additional file 1. [file 12866_2020_1800_MOESM1_ESM.pdf]

BMC Microbiology

## Supplementary Material for:

# Discovery of Thermophilic *Bacillales* using Reduced-representation Genotyping for Identification.

Talamantes-Becerra, B., Carling, J., Kilian, A., Georges, A.

## Sampling sites

## The Great Artesian Basin, South Australia

Photos by Berenice Talamantes-Becerra

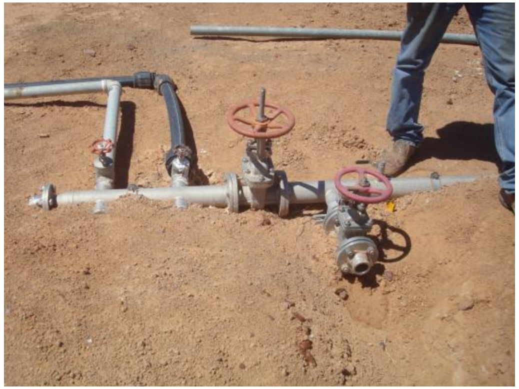

**Clifton Hills Station**

Water temperature:  80 ˚C

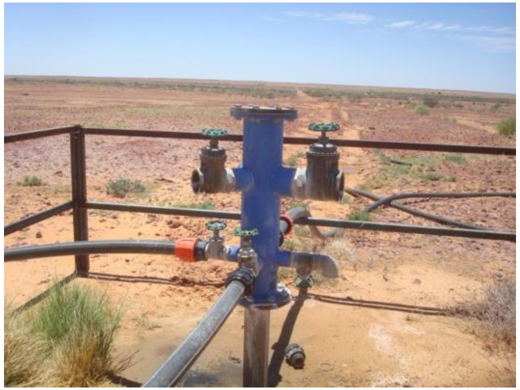

**Mt Gason**

Water temperature: 80 ˚C

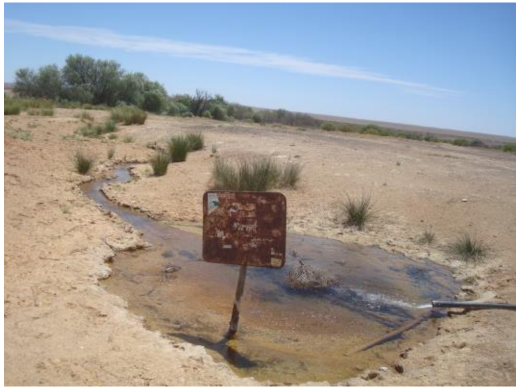

**Mirra Mita**

Water temperature: 79 ˚C

**Mirra Mita:** Eight sediment samples were collected from the bore drain at various distances from the bore head along the temperature gradient.

Photos by Berenice Talamantes-Becerra

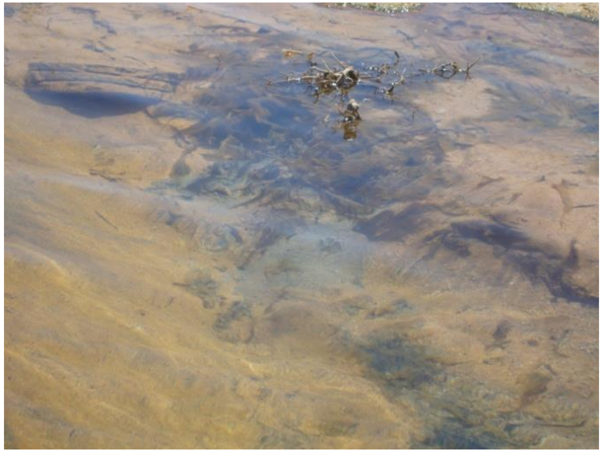

**MMMud1 (Mirra Mita mud sample 1)**

Water temperature: 79 ˚C

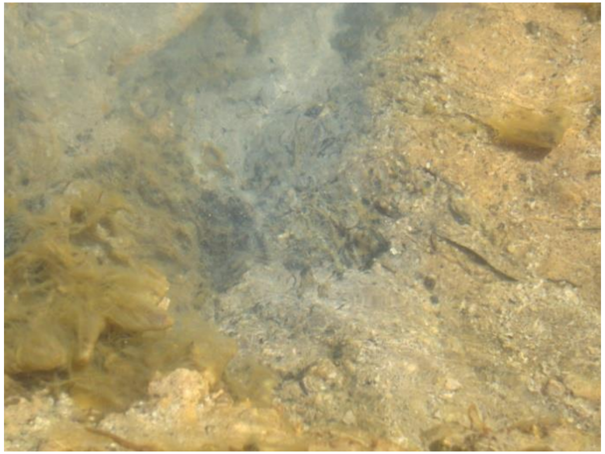

**MMMud2 (Mirra Mita mud sample 2)**

Water temperature: 68 ˚C

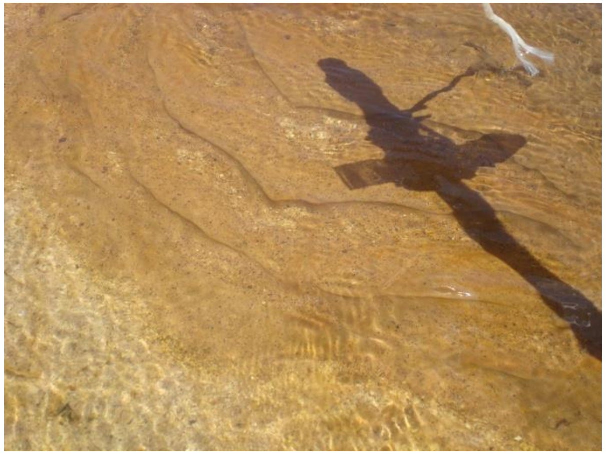

**MMMud3 (Mirra Mita mud sample 3)**

Water temperature: 74 ˚C

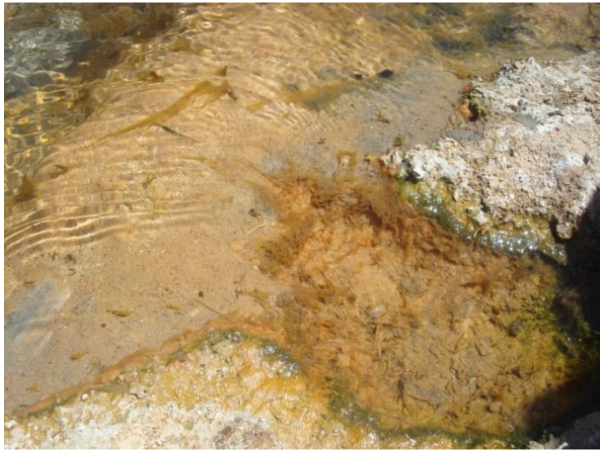

**MMMud4 (Mirra Mita mud sample 4)**

Water temperature: 62 ˚C

Photos by Berenice Talamantes-Becerra

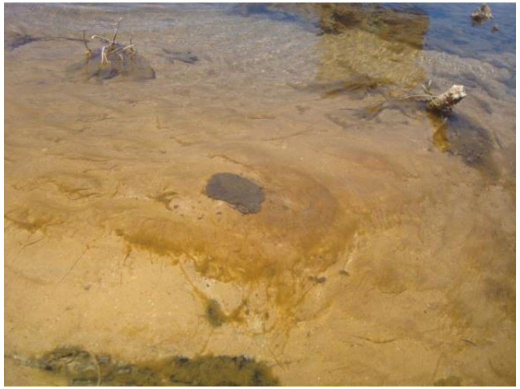

**MMMud5 (Mirra Mita mud sample 5)**

Water temperature:  65-67 ˚C

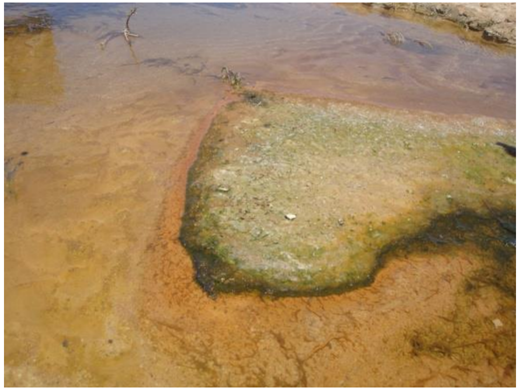

**MMMud6 (Mirra Mita mud sample 6)**

Water temperature: 50 ˚C

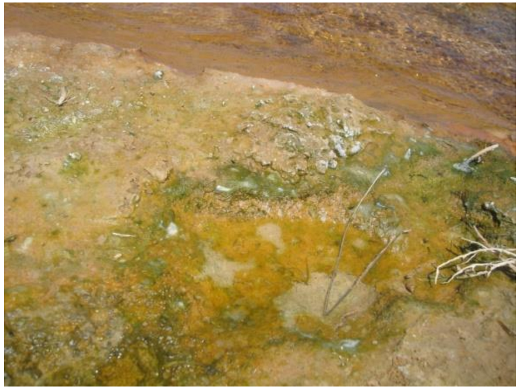

**MMMud7 (Mirra Mita mud sample 7)**

Water temperature: 39 ˚C

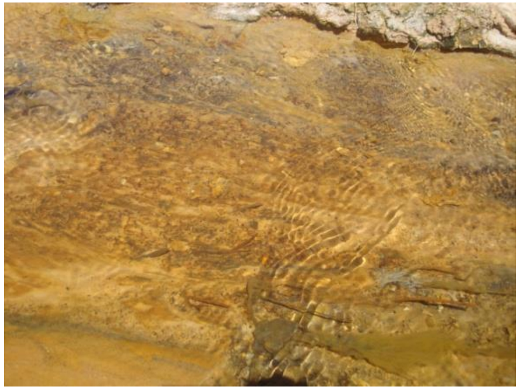

**MMMud8 (Mirra Mita mud sample 8)**

Water temperature: 74 ˚C

Photos by Berenice Talamantes-Becerra

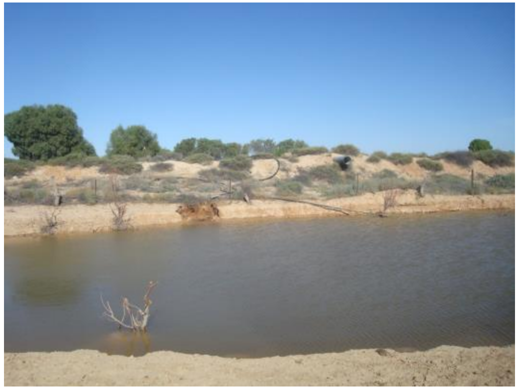

**Mungerannie Station**

Water temperature:  60 ˚C

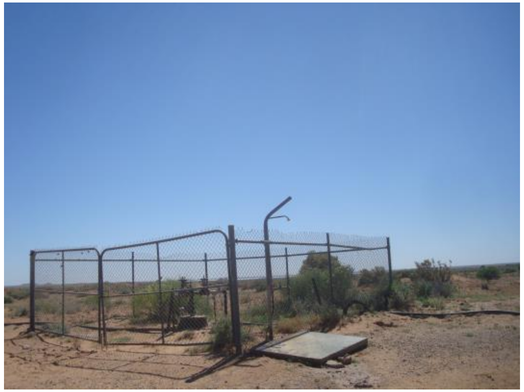

**Lake Harry**

Water temperature: 46 ˚C

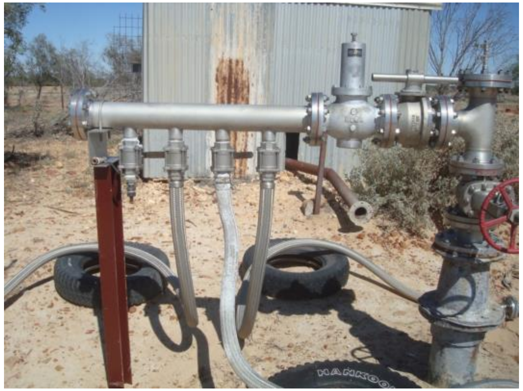

**Kopperamanna**

Water temperature: 60 ˚C

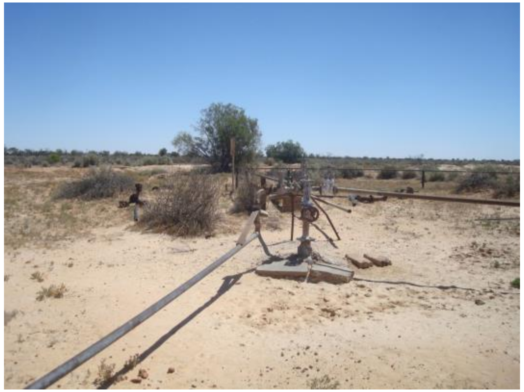

**Etadunna Station**

Water temperature: 77.9 ˚C

Photos by Berenice Talamantes-Becerra

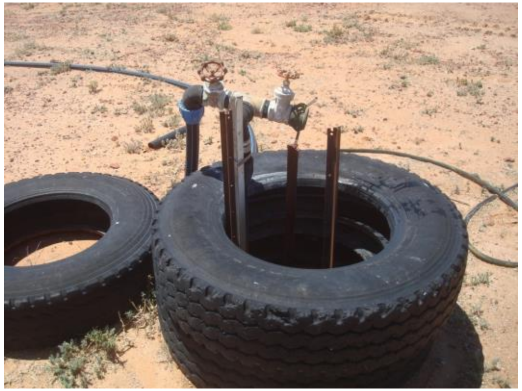

**Dulkaninna Station**

Water temperature:  47.8 ˚C

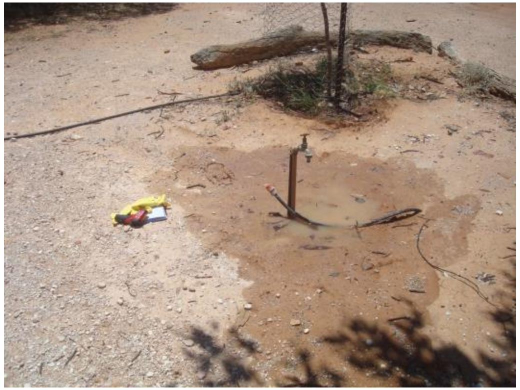

**Clayton station**

Water temperature: 34 ˚C

Photos by Berenice Talamantes-Becerra
